# Supplementary material for: UPF1 plays critical roles in early B cell development
Source: Nat Commun. 2024 Jul 9;15:5765. doi: 10.1038/s41467-024-50032-6 (PMC11233602; doi:10.1038/s41467-024-50032-6)
Supplement: Supplementary file 1 — Supplementary Information [file 41467_2024_50032_MOESM1_ESM.pdf]

# Supplementary Information

## UPF1 Plays Critical Roles in Early B Cell Development


| | |
|---|---|
| **Supplementary Figure 1** | **Analysis and generation of B cell specific Upf1 deficient mice** |
| **Supplementary Figure 2** | **Enrichment score plot of down-regulated genes in Upf1-deficient early LPre-B cells** |
| **Supplementary Figure 3** | **Loss of IFNαR1 signaling does not impact B cell development.** |
| **Supplementary Figure 4** | **Genetic pre-arrangement of *Igh* locus rescued early to late LPre B cell differentiation in *Upf*1-cKO mice** |
| **Supplementary Figure 5** | **Genetic pre-arrangement of *Igh* locus did not rescue the splenic B cell population in *Upf*1-cKO mice** |
| **Supplementary Figure 6** | **Transcriptome analysis of Upf1-cKO/Igh$^{B1\text{-}8hi}$ early LPre- and sPre-B cells** |
| **Supplementary Figure 7** | **Identifying UPF1-target RNAs** |
| **Supplementary Table 1** | **List of primers used for PCR** |
| **Supplementary Table 2** | **List of antibodies** |
| **Supplementary Table 3** | **List of primers used for RT-qPCR** |

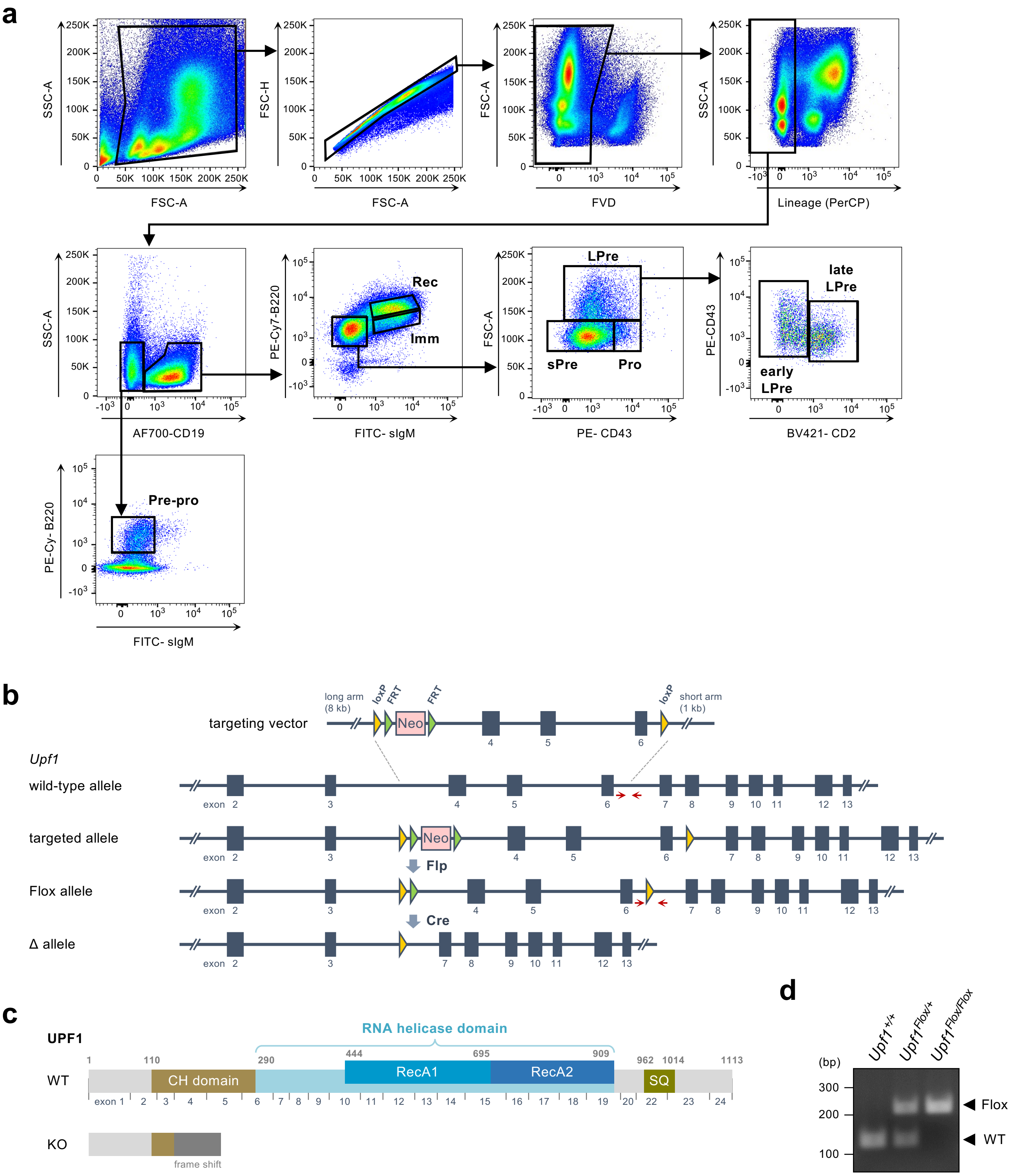


**Supplementary Figure 1 Analysis and generation of B cell specific Upf1 deficient mice**
(**a**) Gating strategy of BM B cells. Harvested cells were stained and separated with the indicated markers. (**b**) Structure of the target *Upf1* allele. Red arrows indicate the primers used for genotyping. (**c**) The schematic illustration of mouse UPF1 and predicted translation product from *Upf1*$^{\mathrm{Flox}}$ allele in *Upf1*-cKO. (**d**) PCR genotyping of genomic DNAs from *Upf1*$^{+/+}$, *Upf1*$^{\mathrm{Flox}/+}$, or *Upf1*$^{\mathrm{Flox/Flox}}$ mice. Results are representative of at least three independent experiments.

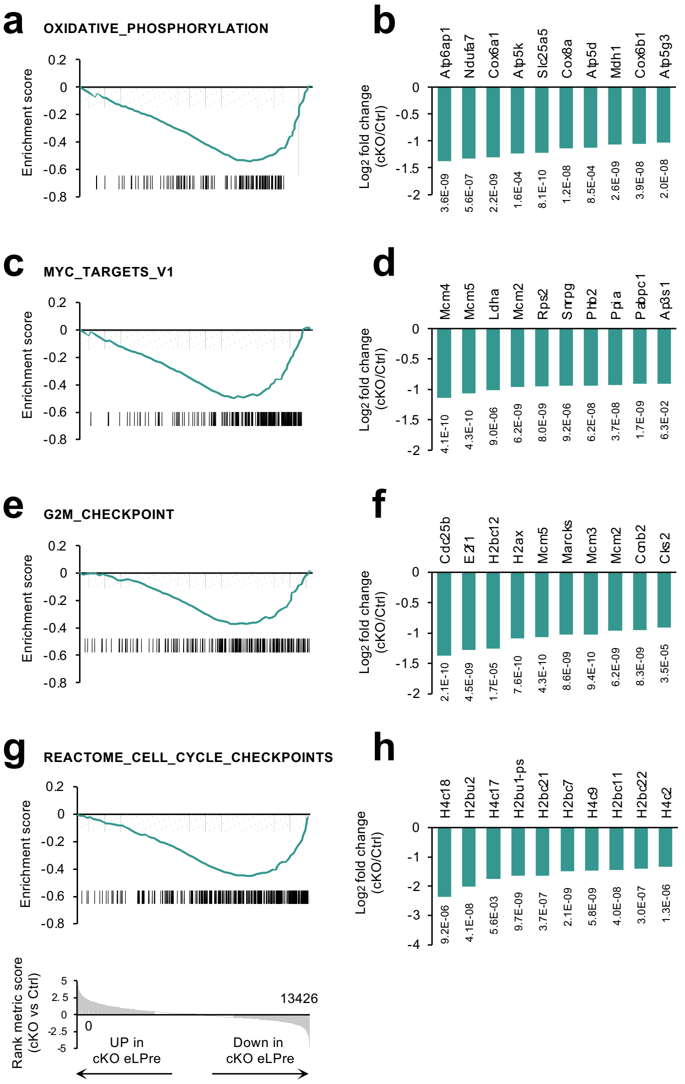


**Supplementary Figure 2 Enrichment score plot of down-regulated genes in Upf1-deficient early LPre-B cells** (**a**-**h**) Enrichment score plot (**a**, **c**, **e**, **g**) and comparison of each gene expression between *Upf1*-cKO- and Ctrl-early LPre-B cells (**b**, **d**, **f**, **h**) in indicated gene sets (**a**, **b**; OXIDATIVE PHOSPHORYLATION, **c**, **d**; MYC TARGETS V1, **e**, **f**; G2M CHECKPOINT, **g**, **h**; CELL CYCLE CHECKPOINTS). Bars indicate Log2FC (Upf1-cKO vs Ctrl) of the top 10 genes with the largest difference between cKO and Ctrl, and the number adjacent to each bar indicates adj.*P*. The statistical analyses were performed using GSEA.

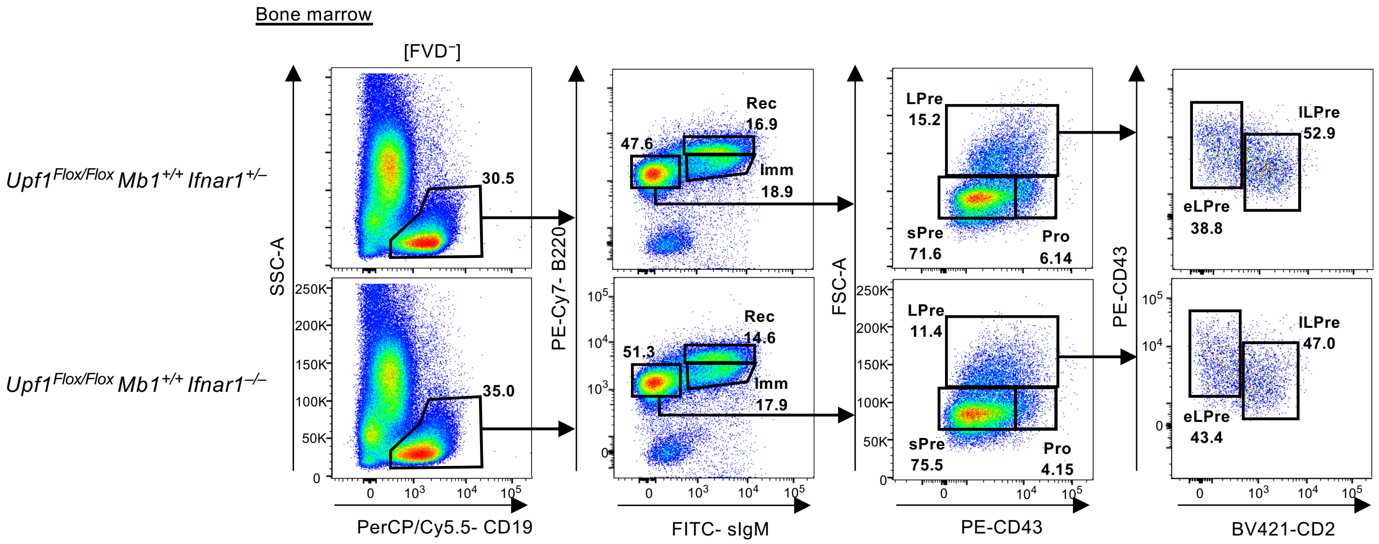


**Supplementary Figure 3 Loss of IFNαR1 signaling does not impact B cell development.**
Flow cytometry plots of indicated populations in the BM of indicated mice. Results are representative of two independent experiments.

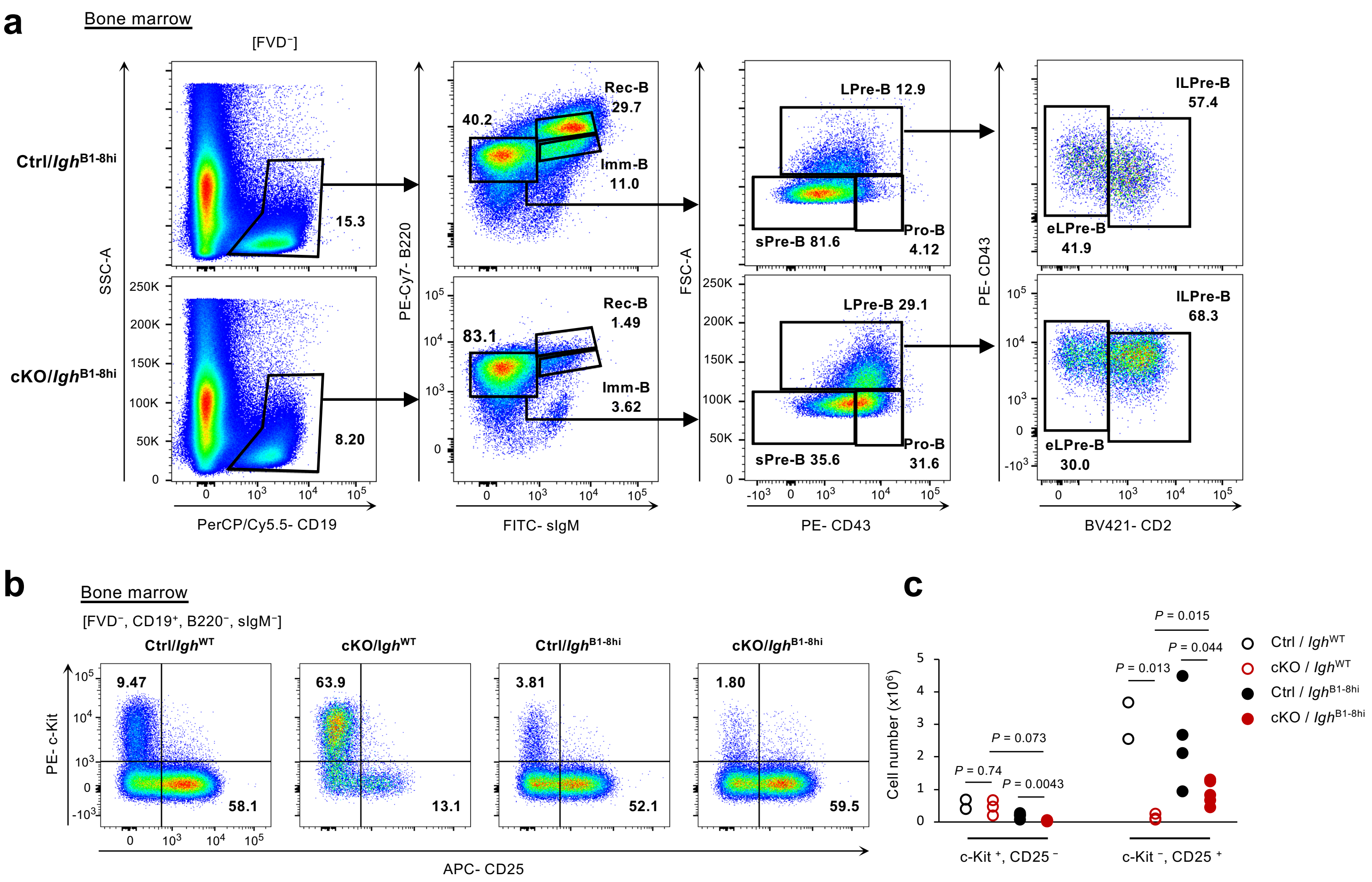


**Supplementary Figure 4 Genetic pre-arrangement of *Igh* locus rescued early to late LPre B cell differentiation in *Upf*1-cKO mice**

(**a**) Flow cytometry of indicated populations in the BM of indicated mice. (**b**, **c**) Flow cytometry plots (**b**) and cell number quantification (**c**) of FVD$^-$, CD19$^+$, B220$^+$, sIgM$^-$ cells derived from BM of indicated mice. BMs were harvested from bones of both hind legs. The *p*-values were calculated using two-sided Student's t-test. Results are representative of at least three independent experiments ($N$ = 3 (Ctrl/*Igh*$^{WT}$, cKO/*Igh*$^{WT}$), $N$ = 4 (Ctrl/*Igh*$^{B1\text{-}8hi}$), $N$ = 5 (cKO/*Igh*$^{B1\text{-}8hi}$).

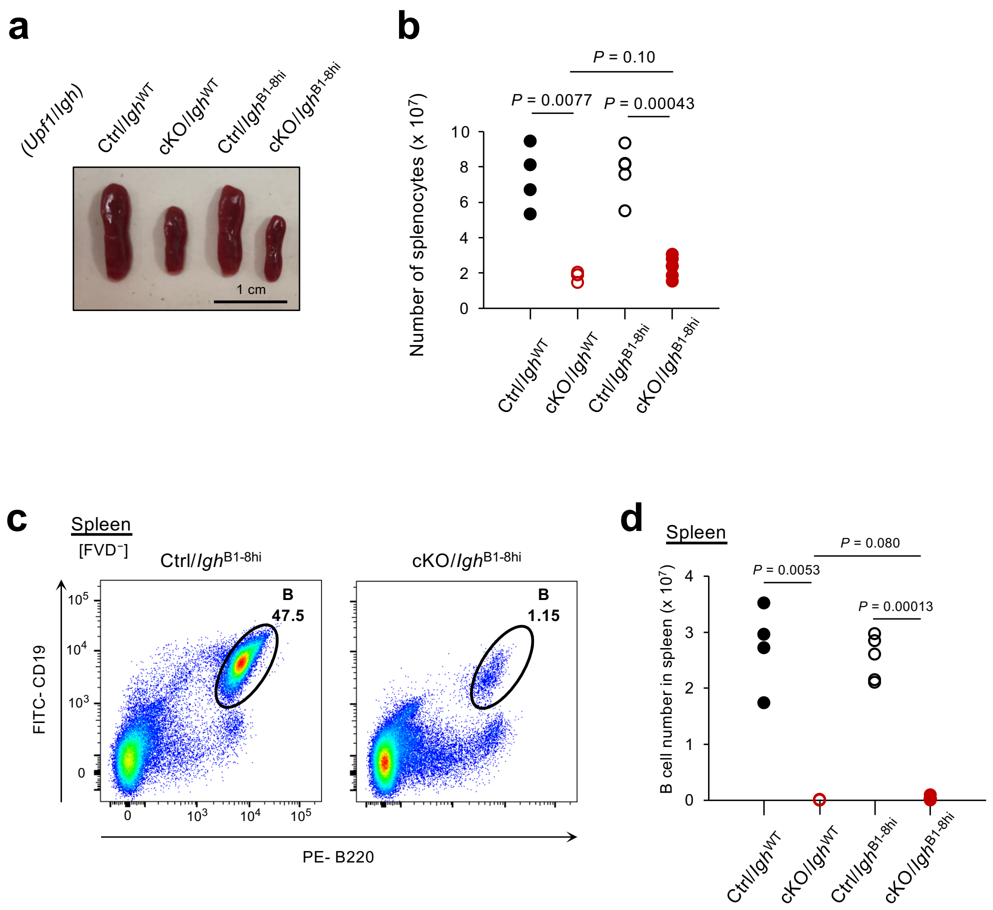


**Supplementary Figure 5 Genetic pre-arrangement of *Igh* locus did not rescue the splenic B cell population in *Upf*1-cKO mice**

(**a**) Appearance of spleens derived from indicated mice. Results are representative of at least three independent experiments. (**b**) Numbers of splenocytes derived from indicated mice. $N = 4$ (Ctrl/$Igh^{\mathrm{WT}}$, cKO/$Igh^{\mathrm{WT}}$), $N = 5$ (Ctrl/$Igh^{\mathrm{B1\text{-}8hi}}$), $N = 6$ (cKO/$Igh^{\mathrm{B1\text{-}8hi}}$). (**c** and **d**) Flow cytometry plots (**c**) and quantifications (**d**) of splenic B cells ($B220^{+}$, $CD19^{+}$) of indicated mice. $N = 4$ (Ctrl/$Igh^{\mathrm{WT}}$ cKO/$Igh^{\mathrm{WT}}$), $N = 5$ (Ctrl/$Igh^{\mathrm{B1\text{-}8hi}}$), $N = 6$ (cKO/$Igh^{\mathrm{B1\text{-}8hi}}$). The *p*-values were calculated using two-sided Student's t-test.

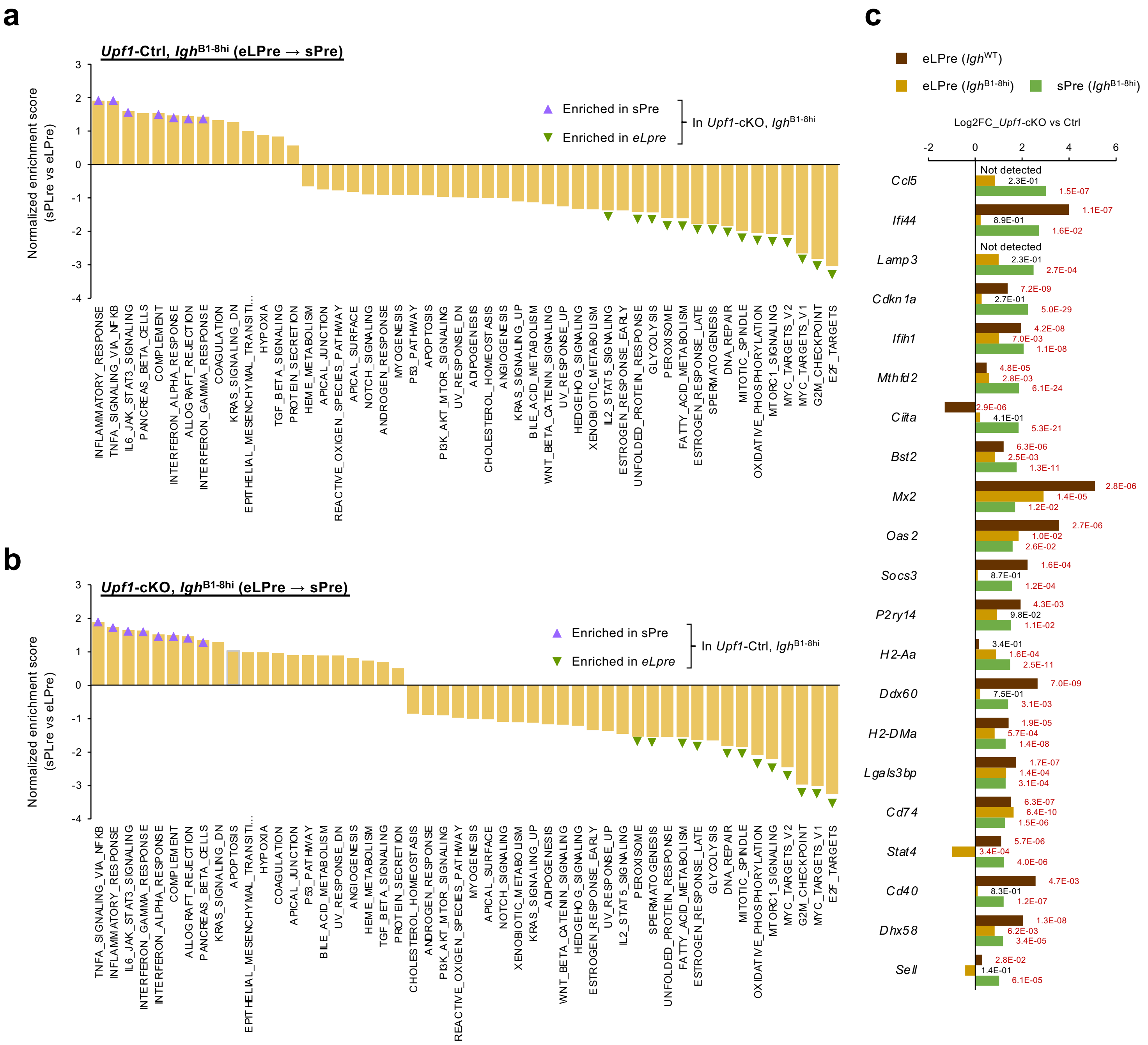


**Supplementary Figure 6 Transcriptome analysis of Upf1-cKO/Igh$^{B1\text{-}8hi}$ early LPre- and sPre-B cells**

(**a, b**) Normalized enrichment score of GSEA (sPre- vs early LPre (eLPre)-B cells) in *Upf1*-Ctrl/*Igh*$^{B1\text{-}8hi}$ (**a**) and *Upf1*-cKO/*Igh*$^{B1\text{-}8hi}$ (**b**). Green and purple arrows indicate the gene sets enriched in early LPre- and sPre-B cells in antagonistic genotype (*Upf1*-Ctrl/*Igh*$^{B1\text{-}8hi}$ for *Upf1*-cKO/*Igh*$^{B1\text{-}8hi}$ and vice versa), respectively. (**c**) Comparison of IFNA/IFNG RESPONSE gene expression between *Upf1*-cKO vs Ctrl in indicated genotypes and cell types. The number adjacent to each bar indicates adj.*P* (red; adj.*P* < 0.05). Only the genes with significantly high expression in *Upf1*-cKO/*Igh*$^{B1\text{-}8hi}$ sPre B cells (Log2FC >= 1, adj.*P* <0.05) are shown. The statistical analyses were performed using limma (*Igh*$^{WT}$), and edgeR (*Igh*$^{B1\text{-}8hi}$).

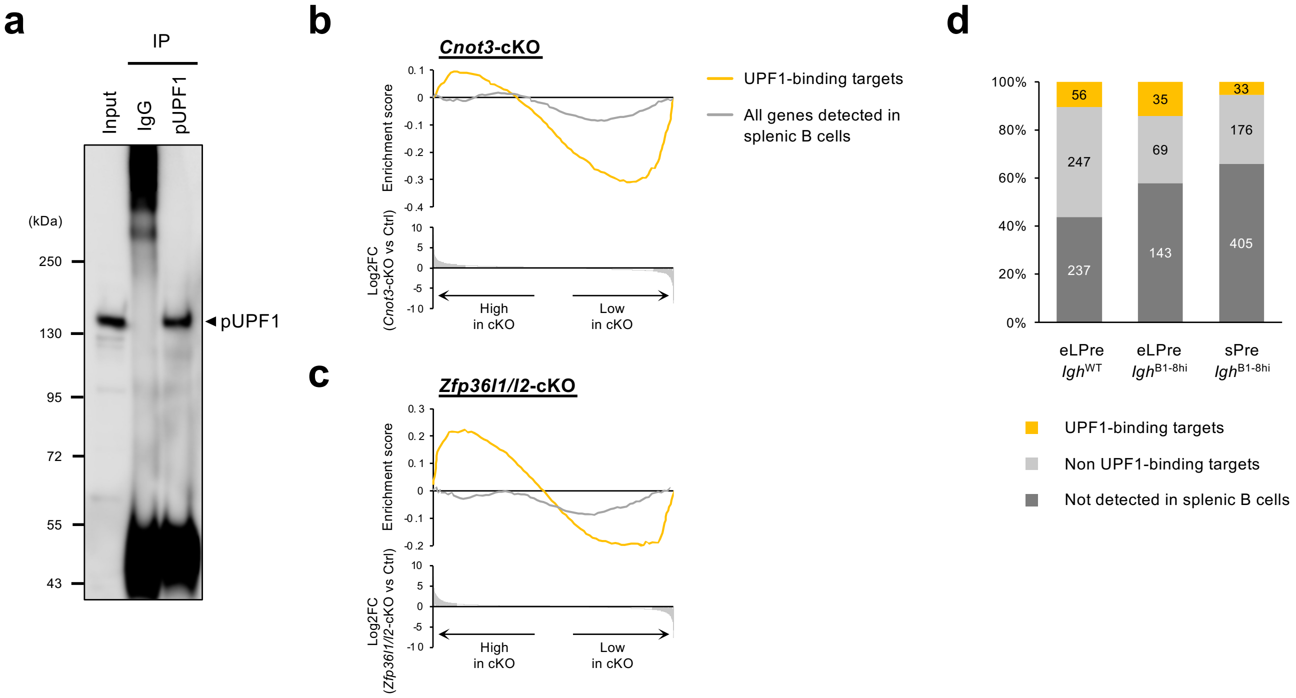


**Supplementary Figure 7 Identifying UPF1-target RNAs**

(**a**) Immunoblot analysis of p-UPF1 precipitated with anti-p-UPF1 antibody or normal rIgG1 (negative control). Arrow indicates p-UPF1. Results are representative of two independent experiments. (**b**, **c**) Enrichment score plot of UPF1-binding targets (yellow) and all genes detected in splenic B cells (gray) in *Cnot3*-cKO vs Ctrl (**b**) and *Zfp36l1/l2*-cKO vs Ctrl (**c**). (**d**) Percentage of detected (yellow; target of UPF1, gray; non-target of UPF1) and undetected (dark gray) RNAs in RIP-seq using splenic B cells among the genes with significantly high expression in *Upf1*-cKO in indicated genotypes and cell types. The absolute number of the genes is shown in each bar.

## Supplementary Table 1 List of primers used for PCR

| | |
|---|---|
| Genotyping for *Upf1*-Flox forward | GAATTGGTGGCATGGGTCCCATATCCCAG |
| Genotyping for *Upf1*-Flox reverse | GACTCTTCCTGGCCTTGAGCCTGGATG |
| $V_H$J558 forward | CGAGCTCTCCARCACAGCCTWCATGCARCTCARC |
| $V_H$7183 forward | CGGTACCAAGAASAMCCTGTWCCTGCAAATGASC |
| $D_H$ forward | TTCAAAGCACAATGCCTGGCT |
| $J_H$3 reverse | GTCTAGATTCTCACAAGAGTCCGATAGACCCTGG |
| Cμ forward | TGGCCATGGGCTGCCTAGCCCGGGACTT |
| Cμ reverse | GCCTGACTGAGCTCACACAAGGAGGA |
| Vk forward | GGCTGCAGSTTCAGTGGCAGTGGRTCWGGRAC |
| Jk reverse | CTCATTCCTGTTGAAGCTCTTGACAATGGG |

## Supplementary Table 2 List of antibodies

| Antibody | Cat# | Company | Clone | Application | Dilution |
|---|---|---|---|---|---|
| anti-Upf1 | NBP1-05967 | Novus biologicals | Polyclonal | Immunoblot | 1:1000 |
| anti-phospho-Upf1 | 07-1016 | Merck | Polyclonal | Immunoblot | 1:1000 |
| anti-β-actin | sc-1615 | Santa Cruz | Polyclonal | Immunoblot | 1:2000 |
| anti-Mouse IgG | NA9310 | Cytiva | Polyclonal | Immunoblot | 1:2000 |
| anti-Rabbit IgG | NA9340 | Cytiva | Polyclonal | Immunoblot | 1:2000 |
| Biotin anti-mouse CD2 | 100103 | Biolegend | RM2-5 | Flow cytometry | 1:300 |
| PerCP/Cy5.5 anti-mouse CD19 | 152405 | Biolegend | 1D3 | Flow cytometry | 1:200 |
| FITC anti-mouse CD19 | 115506 | Biolegend | 6D5 | Flow cytometry | 1:200 |
| Alexa Fluor 700 anti-mouse CD19 | 115527 | Biolegend | 6D5 | Flow cytometry | 1:200 |
| APC Rat Anti-Mouse CD19 | 550992 | BD Pharmingen | 1D3 | Flow cytometry | 1:200 |
| APC anti-mouse CD25 | 102011 | Biolegend | PC61 | Flow cytometry | 1:200 |
| PE Rat Anti-Mouse CD43 | 561857 | BD Pharmingen | S7 | Flow cytometry | 1:200 |
| FITC anti-mouse IgM | 406505 | Biolegend | RMM-1 | Flow cytometry | 1:200 |
| APC anti-mouse IgM | 406509 | Biolegend | RMM-1 | Flow cytometry | 1:200 |
| PE-Cy7 Rat Anti-Mouse CD45R/B220 | 552772 | BD Pharmingen | RA3-6B2 | Flow cytometry | 1:200 |
| FITC anti-mouse/human CD45R/B220 | 103206 | Biolegend | RA3-6B2 | Flow cytometry | 1:200 |
| PE anti-mouse/human CD45R/B220 | 103208 | Biolegend | RA3-6B2 | Flow cytometry | 1:200 |
| PE anti-mouse CD117 (c-Kit) | 105807 | Biolegend | 2B8 | Flow cytometry | 1:200 |
| PerCP Hamster Anti-Mouse CD3e | 553067 | BD Pharmingen | 145-2C11 | Flow cytometry | 1:100 |
| PerCP/Cy5.5 anti-mouse/human CD11b | 101228 | Biolegend | M1/70 | Flow cytometry | 1:100 |
| PerCP anti-mouse CD11c | 117326 | Biolegend | N418 | Flow cytometry | 1:100 |
| PerCP anti-mouse F4/80 | 123125 | Biolegend | BM8 | Flow cytometry | 1:100 |
| PerCP/Cy5.5 anti-mouse TER-119 | 116227 | Biolegend | TER-119 | Flow cytometry | 1:100 |
| Brilliant Violet 421 Streptavidin | 405225 | Biolegend | — | Flow cytometry | 1:400 |

## Supplementary Table 3 List of primers used for RT-qPCR

| | Forward | Reverse |
|---|---|---|
| 18S rRNA | CGGACAGGATTGACAGATTG | CAAATCGCTCCACCAAGTAA |
| mouse Upf1 | GCTGAACTTCGAGGAAGATG | CTTCCTTGCATTTTGCCCTC |
| mouse Gas5 | CAGGTATTAATGGGTCACCTC | CTTCTATTTGAGCCTCCATCC |
| mouse Mx1 | GATCCGACTTCACTTCCAGATGG | CATCTCAGTGGTAGTCAACCC |
| mouse Mx2 | TTCACCAGGCTCCGAAAAGAG | ACAAACCCTGGCAATTCTCG |
