# Supplementary material for: UPF1 plays critical roles in early B cell development
Source: Nat Commun. 2024 Jul 9;15:5765. doi: 10.1038/s41467-024-50032-6 (PMC11233602; doi:10.1038/s41467-024-50032-6)
Supplement: Supplementary file 3 — Description of Additional Supplementary Files [file 41467_2024_50032_MOESM3_ESM.pdf]

Description of Additional Supplementary Files

File Name: Supplementary Data 1
Description: The Data of RNA-seq analysis of early LPre-B cells (Upf1-cKO vs Ctrl)

File Name: Supplementary Data 2
Description: The Data of RNA-seq analysis of early LPre-B (eLPre) and sPre-B cells from *Upf1*-cKO/*Igh*$^{B1\text{-}8hi}$ and Ctrl/*Igh*$^{B1\text{-}8hi}$ mice (eLPre_cKO vs Ctrl, sPre_cKO vs Ctrl, Ctrl_sPre vs eLPre, cKO_sPre vs eLPre)

File Name: Supplementary Data 3
Description: The Data of RIP-seq analysis using anti-p-UPF1 antibody in activated splenic B cells
