# Supplementary material for: UPF1 plays critical roles in early B cell development
Source: Nat Commun. 2024 Jul 9;15:5765. doi: 10.1038/s41467-024-50032-6 (PMC11233602; doi:10.1038/s41467-024-50032-6)
Supplement: Supplementary file 7 — Reporting Summary [file 41467_2024_50032_MOESM7_ESM.pdf]

nature portfolio

Corresponding author(s): Takashi Mino and Osamu Takeuchi

Last updated by author(s): Jun 3, 2024

Reporting Summary

Nature Portfolio wishes to improve the reproducibility of the work that we publish. This form provides structure for consistency and transparency in reporting. For further information on Nature Portfolio policies, see our Editorial Policies and the Editorial Policy Checklist.

Statistics

For all statistical analyses, confirm that the following items are present in the figure legend, table legend, main text, or Methods section.

n/a	Confirmed	
☐	☒	The exact sample size (*n*) for each experimental group/condition, given as a discrete number and unit of measurement
☐	☒	A statement on whether measurements were taken from distinct samples or whether the same sample was measured repeatedly
☐	☒	The statistical test(s) used AND whether they are one- or two-sided<br>*Only common tests should be described solely by name; describe more complex techniques in the Methods section.*
☒	☐	A description of all covariates tested
☐	☒	A description of any assumptions or corrections, such as tests of normality and adjustment for multiple comparisons
☐	☒	A full description of the statistical parameters including central tendency (e.g. means) or other basic estimates (e.g. regression coefficient) AND variation (e.g. standard deviation) or associated estimates of uncertainty (e.g. confidence intervals)
☐	☒	For null hypothesis testing, the test statistic (e.g. *F*, *t*, *r*) with confidence intervals, effect sizes, degrees of freedom and *P* value noted<br>*Give P values as exact values whenever suitable.*
☒	☐	For Bayesian analysis, information on the choice of priors and Markov chain Monte Carlo settings
☒	☐	For hierarchical and complex designs, identification of the appropriate level for tests and full reporting of outcomes
☒	☐	Estimates of effect sizes (e.g. Cohen's *d*, Pearson's *r*), indicating how they were calculated

Our web collection on statistics for biologists contains articles on many of the points above.

Software and code

Policy information about availability of computer code

Data collection	To gain the FACS data, BD FACSDiva were used.
Data analysis	For analysis of obtained FACS data, FlowJo (v10) were used.<br><br>The transcriptome data was analyzed using Galaxy 2 (Afgan et al., 2018). Briefly, identified reads were mapped on the murine genome (mm10) using HISAT2 (single end, unstranded), and the mapped reads were counted using featureCounts.<br><br>Gene Set Enrichment Analysis of Upf1-cKO and Ctrl early LPre-B cells was performed using GSEA software (version 4.3.2). [PMID:16199517 and 12808457]<br>we used all the gene sets and the identified gene sets were visualized using Cytoscape. [PMID: 14597658, 30664679]

For manuscripts utilizing custom algorithms or software that are central to the research but not yet described in published literature, software must be made available to editors and reviewers. We strongly encourage code deposition in a community repository (e.g. GitHub). See the Nature Portfolio guidelines for submitting code & software for further information.

Data

Policy information about availability of data

All manuscripts must include a data availability statement. This statement should provide the following information, where applicable:

- Accession codes, unique identifiers, or web links for publicly available datasets
- A description of any restrictions on data availability
- For clinical datasets or third party data, please ensure that the statement adheres to our policy

The raw RNA sequence data is deposited in GEO (eLPre RNA-seq and RIP-seq: GSE234830, B1-8hi RNA-seq: GSE264655).
===
To review GEO accession GSE234830:
Go to https://www.ncbi.nlm.nih.gov/geo/query/acc.cgi?acc=GSE234830
Enter token cvcviyuobjslfgr into the box

To review GEO accession GSE264655:
Go to https://www.ncbi.nlm.nih.gov/geo/query/acc.cgi?acc=GSE264655
Enter token azeraikgzjetnqd into the box
===
The processed data reported in this paper are provided in the Supplementary Data files. All data supporting the findings of this study are present in the article and/or its Supplementary Information files. Source data are provided with this paper.

Research involving human participants, their data, or biological material

Policy information about studies with human participants or human data. See also policy information about sex, gender (identity/presentation), and sexual orientation and race, ethnicity and racism.

Reporting on sex and gender

Use the terms sex (biological attribute) and gender (shaped by social and cultural circumstances) carefully in order to avoid confusing both terms. Indicate if findings apply to only one sex or gender; describe whether sex and gender were considered in study design; whether sex and/or gender was determined based on self-reporting or assigned and methods used. Provide in the source data disaggregated sex and gender data, where this information has been collected, and if consent has been obtained for sharing of individual-level data; provide overall numbers in this Reporting Summary. Please state if this information has not been collected. Report sex- and gender-based analyses where performed, justify reasons for lack of sex- and gender-based analysis.

Reporting on race, ethnicity, or other socially relevant groupings

Please specify the socially constructed or socially relevant categorization variable(s) used in your manuscript and explain why they were used. Please note that such variables should not be used as proxies for other socially constructed/relevant variables (for example, race or ethnicity should not be used as a proxy for socioeconomic status). Provide clear definitions of the relevant terms used, how they were provided (by the participants/respondents, the researchers, or third parties), and the method(s) used to classify people into the different categories (e.g. self-report, census or administrative data, social media data, etc.) Please provide details about how you controlled for confounding variables in your analyses.

Population characteristics

Describe the covariate-relevant population characteristics of the human research participants (e.g. age, genotypic information, past and current diagnosis and treatment categories). If you filled out the behavioural & social sciences study design questions and have nothing to add here, write "See above."

Recruitment

Describe how participants were recruited. Outline any potential self-selection bias or other biases that may be present and how these are likely to impact results.

Ethics oversight

Identify the organization(s) that approved the study protocol.

Note that full information on the approval of the study protocol must also be provided in the manuscript.

Field-specific reporting

Please select the one below that is the best fit for your research. If you are not sure, read the appropriate sections before making your selection.

☒ Life sciences ☐ Behavioural & social sciences ☐ Ecological, evolutionary & environmental sciences

For a reference copy of the document with all sections, see nature.com/documents/nr-reporting-summary-flat.pdf

Life sciences study design

All studies must disclose on these points even when the disclosure is negative.

Sample size

No statistical methods were used to predetermine the sample size. Sample size were determined based on feasibility, pilot experiments, and according to published iterature.

Data exclusions | No data were excluded from analyses.

Replication | Mouse and in vitro experiments were conducted and successfully replicated with sufficient numbers of mice or biological replicates as indicated in the Figure Legends section. All experiments were successfully repeated at least two times

Randomization | Randomization was not required in this study because there is no statistic that requires randomization of samples. For all animal experiments, control or knock-out mice were randomly selected from the littermates. For other experiments, all samples were treated in the same way to decrease the variability.

Blinding | The experiments were not performed blindly due to the researchers needed to verify the genotypes of mice.

Reporting for specific materials, systems and methods

We require information from authors about some types of materials, experimental systems and methods used in many studies. Here, indicate whether each material, system or method listed is relevant to your study. If you are not sure if a list item applies to your research, read the appropriate section before selecting a response.

Materials & experimental systems

n/a	Involved in the study
☐	☒ Antibodies
☒	☐ Eukaryotic cell lines
☒	☐ Palaeontology and archaeology
☐	☒ Animals and other organisms
☒	☐ Clinical data
☒	☐ Dual use research of concern
☒	☐ Plants

Methods

n/a	Involved in the study
☒	☐ ChIP-seq
☐	☒ Flow cytometry
☒	☐ MRI-based neuroimaging

Antibodies

Antibodies used

Primary antibodies for immunoblot analysis used in this study were as follows: anti-Upf1 (Novus biologicals, NBP1-05967), anti-phospho-Upf1(Merck, 07-1016), anti-β-actin (sc-1615, Santa Cruz).

Secondary HRP-conjugated antibodies were from Cytiva (anti-Mouse IgG, HRP-Linked F(ab')2 Fragment Sheep, NA9310, and anti-Rabbit IgG, HRP-Linked F(ab')2 Fragment Donkey, NA9340).

Antibodies and fluorescent dyes for flow cytometry analysis were as follows:
Biotin anti-mouse CD2 (Biolegend, 100103, clone: RM2-5), PerCP/Cy5.5 anti-mouse CD19 (Biolegend, 152405, clone: 1D3), FITC anti-mouse CD19 (Biolegend, 115506, clone: 6D5), Alexa Fluor 700 anti-mouse CD19 (Biolegend, 115527, clone: 6D5), APC Rat Anti-Mouse CD19 (BD Pharmingen, 550992, clone: 1D3), APC anti-mouse CD25 (Biolegend, 102011, clone: PC61), PE Rat Anti-Mouse CD43 (BD Pharmingen, 561857, clone: S7), FITC anti-mouse IgM (Biolegend, 406505, clone: RMM-1), APC anti-mouse IgM (Biolegend, 406509, clone: RMM-1), PE-Cy7 Rat Anti-Mouse CD45R/B220 (BD Pharmingen, 552772, clone: RA3-6B2), FITC anti-mouse/human CD45R/B220 (Biolegend, 103206, clone: RA3-6B2), PE anti-mouse/human CD45R/B220 (Biolegend, 103208, clone: RA3-6B2), PE anti-mouse CD117 (c-Kit) (Biolegend, 105807, clone: 2B8), PerCP Hamster Anti-Mouse CD3e (BD Pharmingen, 553067, clone: 145-2C11), PerCP/Cy5.5 anti-mouse/human CD11b (Biolegend, 101228, clone: M1/70), PerCP anti-mouse CD11c (Biolegend, 117326, clone: N418), PerCP anti-mouse F4/80 (Biolegend, 123125, clone: BM8), PerCP/Cy5.5 anti-mouse TER-119 (Biolegend, 116227, clone: TER-119), Brilliant Violet 421 Streptavidin (Biolegend, 405225).

Validation

All antibodies were validated for the indicated applications by well-known suppliers as follows:

anti-Upf1 (Novus biologicals, NBP1-05967)
https://www.novusbio.com/products/rent1-upf1-hupf1-antibody_nbp1-05967
anti-phospho-Upf1(Merck, 07-1016)
merckmillipore.com/JP/ja/product/Anti-phospho-Upf1-Ser1127-Antibody,MM_NF-07-1016
anti-β-actin (sc-1615, Santa Cruz)
https://www.scbt.com/ja/p/actin-antibody-c-11

Biotin anti-mouse CD2 (Biolegend, 100103, clone: RM2-5)
https://www.biolegend.com/ja-jp/products/biotin-anti-mouse-cd2-antibody-471?GroupID=BLG10658
PerCP/Cy5.5 anti-mouse CD19 (Biolegend, 152405, clone: 1D3)
https://www.biolegend.com/ja-jp/products/percp-cyanine5-5-anti-mouse-cd19-antibody-13640
FITC anti-mouse CD19 (Biolegend, 115506, clone: 6D5)
https://www.biolegend.com/ja-jp/products/fitc-anti-mouse-cd19-antibody-1528?GroupID=BLG10556
Alexa Fluor 700 anti-mouse CD19 (Biolegend, 115527, clone: 6D5)
https://www.biolegend.com/ja-jp/products/alexa-fluor-700-anti-mouse-cd19-antibody-3391
APC Rat Anti-Mouse CD19 (BD Pharmingen, 550992, clone: 1D3)
https://www.bdbiosciences.com/en-ca/products/reagents/flow-cytometry-reagents/research-reagents/single-color-antibodies-ruo/apc-rat-anti-mouse-cd19.550992

APC anti-mouse CD25 (Biolegend, 102011, clone: PC61)
https://www.biolegend.com/ja-jp/products/apc-anti-mouse-cd25-antibody-420
PE Rat Anti-Mouse CD43 (BD Pharmingen, 561857, clone: S7),
https://www.bdbiosciences.com/en-eu/products/reagents/flow-cytometry-reagents/research-reagents/single-color-antibodies-ruo/pe-rat-anti-mouse-cd43.561857
FITC anti-mouse IgM (Biolegend, 406505, clone: RMM-1)
https://www.biolegend.com/ja-jp/products/fitc-anti-mouse-igm-2334?GroupID=BLG3548
APC anti-mouse IgM (Biolegend, 406509, clone: RMM-1)
https://www.biolegend.com/ja-jp/products/apc-anti-mouse-igm-2335?GroupID=BLG3548
PE-Cy7 Rat Anti-Mouse CD45R/B220 (BD Pharmingen, 552772, clone: RA3-6B2)
https://www.bdbiosciences.com/ja-jp/products/reagents/flow-cytometry-reagents/research-reagents/single-color-antibodies-ruo/pe-cy-7-rat-anti-mouse-cd45r-b220.552772
FITC anti-mouse/human CD45R/B220 (Biolegend, 103206, clone: RA3-6B2)
https://www.biolegend.com/ja-jp/products/fitc-anti-mouse-human-cd45r-b220-antibody-445
PE anti-mouse/human CD45R/B220 (Biolegend, 103208, clone: RA3-6B2)
https://www.biolegend.com/ja-jp/products/pe-anti-mouse-human-cd45r-b220-antibody-447?GroupID=GROUP658
PE anti-mouse CD117 (c-Kit) (Biolegend, 105807, clone: 2B8)
https://www.biolegend.com/ja-jp/products/pe-anti-mouse-cd117-c-kit-antibody-75
PerCP Hamster Anti-Mouse CD3e (BD Pharmingen, 553067, clone: 145-2C11)
https://www.bdbiosciences.com/ja-jp/products/reagents/flow-cytometry-reagents/research-reagents/single-color-antibodies-ruo/percp-hamster-anti-mouse-cd3e.553067
PerCP/Cy5.5 anti-mouse/human CD11b (Biolegend, 101228, clone: M1/70)
https://www.biolegend.com/ja-jp/products/percp-cyanine5-5-anti-mouse-human-cd11b-antibody-4257
PerCP anti-mouse CD11c (Biolegend, 117326, clone: N418)
https://www.biolegend.com/ja-jp/products/percp-anti-mouse-cd11c-antibody-4259
PerCP anti-mouse F4/80 (Biolegend, 123125, clone: BM8)
https://www.biolegend.com/ja-jp/products/percp-anti-mouse-f4-80-antibody-4302?GroupID=BLG5319
PerCP/Cy5.5 anti-mouse TER-119 (Biolegend, 116227, clone: TER-119)
https://www.biolegend.com/ja-jp/products/percp-cyanine5-5-anti-mouse-ter-119-erythroid-cells-antibody-4292
Brilliant Violet 421 Streptavidin (Biolegend, 405225)
https://www.biolegend.com/ja-jp/products/brilliant-violet-421-streptavidin-7297?GroupID=GROUP23

Animals and other research organisms

Policy information about studies involving animals; ARRIVE guidelines recommended for reporting animal research, and Sex and Gender in Research

Laboratory animals	Mice of both sexes (Upf1-floxed, Ifnar1-deficient, B1-8high knock-in mice) were from 8 to 16 weeks of age. C57BL/6JJcl strains were used. The strains used in this study were described in the Methods section. Mice were maintained at Kyoto University animal facilities with a 12 h light/dark cycle and access to food and water ad libitum. Room temperature was maintained at 23 ± 3 °C, with a relative humidity of 50 ± 20%.
Wild animals	This study does not involve wild animals
Reporting on sex	Both sexes mice were included in this study.
Field-collected samples	This study does not involve field-collected samples.
Ethics oversight	All animal experiments were conducted in compliance with the regulations approved by the Committee for Animal Experiments of the Institute for Frontier Life and Medical Sciences and Graduate School of Medicine, Kyoto University.

Note that full information on the approval of the study protocol must also be provided in the manuscript.

Flow Cytometry

Plots

Confirm that:

☒ The axis labels state the marker and fluorochrome used (e.g. CD4-FITC).

☒ The axis scales are clearly visible. Include numbers along axes only for bottom left plot of group (a 'group' is an analysis of identical markers).

☒ All plots are contour plots with outliers or pseudocolor plots.

☒ A numerical value for number of cells or percentage (with statistics) is provided.

Methodology

Sample preparation	Mouse bone marrow cells or splenocytes were collected and single cell suspension was prepared following standard procedures. The detail of the procedures of sample preparation are described in the Methods section.

Instrument	FACSVerse (BD Biosciences), FACSAriaII (BD Biosciences), FACSAriaIII (BD Biosciences), or LSRFortessa X-20 (BD Biosciences)
Software	FlowJo (v10)
Cell population abundance	Purity was determined by flow cytometry of the sorted population in the pilot experiments.
Gating strategy	For all experiments, cells were identified by FSC-A/SSC-A, followed by singlet isolation based on FSC-A/FSC-H doublet-exclusion gating. Fixable Viability Dye positive cells were excluded as dead cells. Detailed gating strategies for specific cell populations were provided in the figures.

☒ Tick this box to confirm that a figure exemplifying the gating strategy is provided in the Supplementary Information.
